# Tumor Cell-associated Exosomes Robustly Elicit Anti-tumor Immune Responses through Modulating Dendritic Cell Vaccines in Lung Tumor

**DOI:** 10.7150/ijbs.38414

**Published:** 2020-01-14

**Authors:** Ce Wang, Xue Huang, Yingjuan Wu, Jingbo Wang, Furong Li, Guoqing Guo

**Affiliations:** 1 Department of anatomy, School of Medicine, Jinan University, Guangzhou 510632, China; 2 Shenzhen Key Laboratory of Stem cell research and clinical transformation, Guangdong Engineering Technology Research Center of Stem cell and Cell therapy, Translational Medicine Collaborative Innovation Center, The Second Clinical Medical College (Shenzhen People's Hospital), Jinan University, Shenzhen 518020, China

**Keywords:** tumor cell-associated exosome, immune response, DC vaccine, immunosuppression, lung tumor

## Abstract

DC vaccine-based immunotherapy is emerging as a novel therapeutic strategy for cancer treatment, however, antitumor effect of DC vaccines based on tumor cell lysates (TCLs) remains unsatisfactory due to poor immunogenicity of tumor antigens. Although tumor-associated exosomes (TAEs) have been reported as a promising antigen for DC vaccines, it remains unclear how TAE-based DC vaccine induced antitumor immunity in lung cancer.

**Methods**: In the present study, we extracted TAEs from the supernatant of tumor cell culture medium, and compared the effect of TAEs with TCLs on DCs. To further evaluate the therapeutic effect of DC_TAE_, we used immunofluorescence and flow cytometry to evaluate the apoptosis of tumor tissue, tumor-infiltrating CD8^+^ T cells and Tregs in TDLNs and spleen. Then the levels of cytokines of IL-12, IFN-γ, L-10 and TGF-β were quantified by ELISA assays.

**Results**: Our data showed that TAEs were more potent than TCLs to promote DC maturation and enhance MHC cross presentation, which directly contributed to more robust tumor-specific cytotoxic T lymphocyte (CTL) response. More importantly, TAEs reduced the expression of PD-L1 of DCs, thereby led to down-regulated population of Tregs *in vitro*. Moreover, DC_TAE_ remarkably suppressed the tumor growth and prolonged survival rate *in vivo*, due to participance of CD8^+^ T cells and decreased Tregs in TDLNs and spleen.

**Conclusion**: TAEs could serve to improve vaccine-elicited immunotherapy by triggering stronger DC-mediated immune responses and decreasing Tregs in the tumor microenvironment.

## Introduction

As we all know, lung cancer is a primary threat to human health, which attracts significant attention due to its aggressive nature, high mortality rate, and low response rates to clinical treatments. The five-year survival rate of lung cancer patients was just 5-15% 1. Therefore, how to trigger a lasting anti-tumor effect and avoid recurrence after treatment are still bottleneck problems to be solved urgently 2.

Dendritic cells (DCs) were the most potent antigen presenting cells that play an essential role in initiating and regulating tumor-specific immune responses. DC-based immunotherapy has been considered as a promising therapeutic strategy for cancer treatment and demonstrated some clinical benefits 3. However, its antitumor effect has been unsatisfactory due to poor immunogenicity of tumor antigens 4-5, low uptake efficiency of antigen 6 and activation of regulatory T cells 7.

Recently, exosomes, as a class of nanoscale membrane vesicles from cells, effective to transfer proteins, lipids and RNA between cells, has garnered considerable interest 8. Compared with the synthesis of nanoparticles, exosomes have better biocompatibility and biodegradability 9. At present, exosomes have been extensively studied for diagnostic purposes and as drug delivery vehicles 10-11. In addition, tumor cell-associated exosomes (TAEs) can efficiently deliver a variety of tumor antigens to DCs 12, so they can be used as self-antigen carriers for tumor immunotherapy.

In addition, exosomes of lung cancer cell associated antigens stimulated-DCs activated CD4^+^ T and CD8^+^ T lymphocytes to induce anti-tumor immune response 13. The CD40 ligand modified exosome of lung cancer cells have activated DCs effectively, inhibited the progress of lung cancer and prolonged the survival time of mice 14. The exosomes of non-small cell lung cancer cells with high Rab-27a expression also effectively stimulated the proliferation and maturation of DCs, subsequently significantly increased the proliferation of CD4^+^ T cells, playing an immunoregulatory role 15. In addition, the exosomes from hepatocellular carcinoma cells contain tumor-specific antigens, which have improved the overall survival rate of mice with hepatocellular carcinoma by activating the anti-tumor immune response 16.

Although TAEs have been reported as a promising antigen for DC vaccines 16-17, it remains unclear how TAE-based DC vaccine induced antitumor immunity in lung cancer. In addition, the immune tolerance was also an important challenge for tumor immunotherapy. Regulatory T cells (Tregs) in the tumor microenvironment, especially in TDLNs, play a key role in tumor progression and tumor immune escape 18. Previous studies have shown that TAEs pulsed DC-treated mice reduced numbers of Tregs in HCC tumor tissues compared with the DC and PBS treatment groups 16. These data suggested that developing DC_TAE_ vaccine delivery hold a great potential for improving cancer vaccine efficacy in lung cancer.

In the present study, we examined the feasibility and functionality of TAEs to stimulate the immune response in lung cancer. Our study demonstrated that TAEs were more potent than TCLs to promote DC maturation and MHC cross presentation, which directly contributed to more robust tumor-specific cytotoxic T lymphocyte (CTL) response. More importantly, TAEs reduced the expression of PD-L1 of DCs, contributing to down-regulated population of Tregs *in vitro*. Notably, DC_TAE_ effectively abrogated immunosuppression in the tumor immune microenvironment by decreasing Tregs in TDLNs and spleen, thereby leading to dramatic tumor regression and prolonged survival time. Consequently, TAEs showed superiority over TCLs for DC mediated immunotherapy in lung cancer.

## Materials and Methods

### Materials

Recombinant human/mouse GM-CSF, IL-4 and IL-2 were obtained from Peprotech (CA, USA), human/mouse IL-12, IL-5 and IFN-γ ELISA kits were purchased from Biolegend (CA, USA). Fluorochrome-labeled anti-mouse monoclonal antibodies and anti-human monoclonal antibodies were purchased from eBiosciences (CA, USA). A549 cells and LLC cells were acquired from Shanghai Institutes for Biological Sciences Cell Bank (Shanghai, China). Dulbecco's Modified Eagle Medium (DMEM high glucose) and fetal bovine serum (FBS) were purchased from Hyclone (CA, USA). Six-week-old female C57BL/6 mice were purchased from Guangdong Province Laboratory Animal Center (Guangzhou, China), and maintained in the institutional animal care facility. All animal protocols were approved by Institutional Animal Care and Usage Committee of Shenzhen People's Hospital.

### Preparation of exosomes and cell lysates

A549 cells (1×10^7^/ml) or LLC cells (1×10^7^/ml) derived from cell culture medium was sequentially centrifuged at 1,000g for 10 minutes, followed by 10,000g for 30 minutes. The supernatant was collected and filtered with a 0.22-µm filter (Millex, Germany), followed by ultracentrifugation at 100,000g for 1 hour to get exosome pellets. Exosome pellets were washed in large volume of phosphate-buffered saline (PBS) and recovered by centrifugation at 100,000g for 1 hour.

To prepare tumor cell lysate (TCL), A549 cells (1×10^7^/ml) or LLC cells (1×10^7^/ml) were subjected to 4 freeze-thaw cycles, followed by sonication for 10 min. The cell lysates were then spun at 13000 rpm for 20 min, and supernatants were collected and filtered with a 0.22 µm filter as tumor Ag. The total protein concentration of exosomes or cell lysates were quantified by the Bradford assay.

### Culture and stimulation of dendritic cells

To prepare the human monocyte-derived dendritic cells (DCs), human monocytes were enriched by plastic adherence of peripheral blood mononuclear cells (PBMCs) in a 100 mm Petri dish at 37 ℃, 5% CO_2_. After 2 h of incubation, the nonadherent cells were removed, and the percentage of CD14^+^ monocytes in adherent cells was over 80%. The enriched monocytes were then cultured in X-vivo 15 medium (Lonza, MD, USA) supplemented with 50 ng/mL of GM-CSF and 50 ng/mL of IL-4 for 5 days to generate immature DCs.

Mouse bone marrow-derived DCs (BMDCs) were generated from mouse bone marrow cells according to a previous report with slight modification 19. Briefly, bone marrow was isolated from the femurs of 6-week-old C57BL/6 mice. After red blood cell lysis, bone marrow cells were cultured in a 60 mm Petri dish overnight. On the next day, the non-adherent cells were carefully harvested and re-suspended in X-vivo 15 medium supplemented with 20 ng/mL of GM-CSF and 10 ng/mL of IL-4.

The medium was changed every other day, and on day 6 the percentage of CD11c^+^ cells was over 80%, as verified by flow cytometry. Human or mouse DCs were treated with 10 µg/mL of different antigens for 24 h, then the cells and culture supernatants were harvested for DC phenotypic analysis and cytokine quantification, respectively.

### Tumor implantation and animal immunization

Six-week C57BL/6J mice were subcutaneously (s.c.) injected with LLC cells (5×10^6^ cells/ mouse) on right buttock. On day 7 after tumor cells implantation, mice were i.v. injected with PBS, antigens, or different cancer vaccines once a week for 3 weeks. Tumor diameters were measured in two dimensions every three days using a caliper, and the tumor volume was calculated according to the following formula: volume (mm^3^) = (width)^2^ × (length) × 1/2. At the end of experiments, tumor tissues were weighted and snap-frozen for immunofluorescent staining.

To determine the effect of DC vaccines on immune cell populations *in vivo*, the spleen and tumor-draining lymph nodes (TDLN) were removed 3-4 days after the last immunization, and cell suspensions were labeled with PE-anti mouse CD8 to identify CD8^+^ T cell.

### Immunofluorescent staining and TUNEL assay

LLC tumors were removed from mice, snap frozen, and then cut into 8 μm-thick cryosections as described previously 19. The cryosections were mounted onto Superfrost Plus glass slides (Fisher Scientific, Houston, TX) and fixed with ice-cold acetone for 10 min. Cell apoptosis in tumor samples was determined by TUNEL assay (Promega, WI, USA) according to manufacturer's instruction. To investigate tumor-infiltrating CD8^+^ T cells, sections were blocked with PBS containing 1% BSA for 1 h, followed by incubation with PE-anti-mouse CD8 (1:500, eBiosciences, USA) at room temperature for 2 h, respectively. After wash, coverslips were applied on the sections with anti-fade mounting medium (Vector Laboratories, CA, USA), and fluorescent images were recorded using a confocal laser scanning microscopy (Leica, Germany).

### Tumor-specific CTL response *in vitro*

Splenocytes were isolated from mice 7-10 days after last immunization and re-stimulated with 100 μg/ml of tumor Ag in the presence of IL-2 (20 U/ml) for 72 h to acquire CTL effectors. The effectors and target cells (LLC cells) were cultured in 96-well plates at various effector / target (E: T) ratios for 6 h. Tumor-specific lysis was quantified using CytoTox 96 Non-Radioactive Cytotoxicity Assay Kit (Promega, WI, USA) according to manufacturer's instruction. In some experiments, splenocytes were re-stimulated with tumor Ag (100 μg/ml) for 72 h, and the production of tumor-specific IFN-γ in supernatants were determined using ELISA kit.

### Western blot analysis

Total protein was extracted from exosomes as described previously 19. Equal amount of cellular protein (80 μg) was resolved on a 12% SDS-PAGE and then transferred onto a polyvinylidene difluoride membrane. The membranes were blocked with 5% non-fat milk in Tris-buffered saline for 1 h and then incubated with mouse anti-CD81, anti-TSG101, anti-CD63, anti-HSP70 and anti-CA125 (Cell Signaling Technology, MA, USA) at 4℃, overnight. After wash, the membranes were incubated with horseradish peroxidase conjugated anti-mouse secondary antibody (KPL, MD, USA) for 1 h. The peroxidase activity associated with the protein bands was detected by enhanced chemiluminescence using ECL (Thermo Scientific, Germany) followed by autoradiograph.

### Statistical Analysis

Data are reported as mean ± SE. The differences among groups were determined using One-way ANOVA analysis followed by Tukey's post test (Graphpad Prism**,** GraphPad Software, La Jolla, CA).

## Results and Discussion

### Formulation and characterization of tumor cell associated exosomes (TAEs)

**Figure 1:**
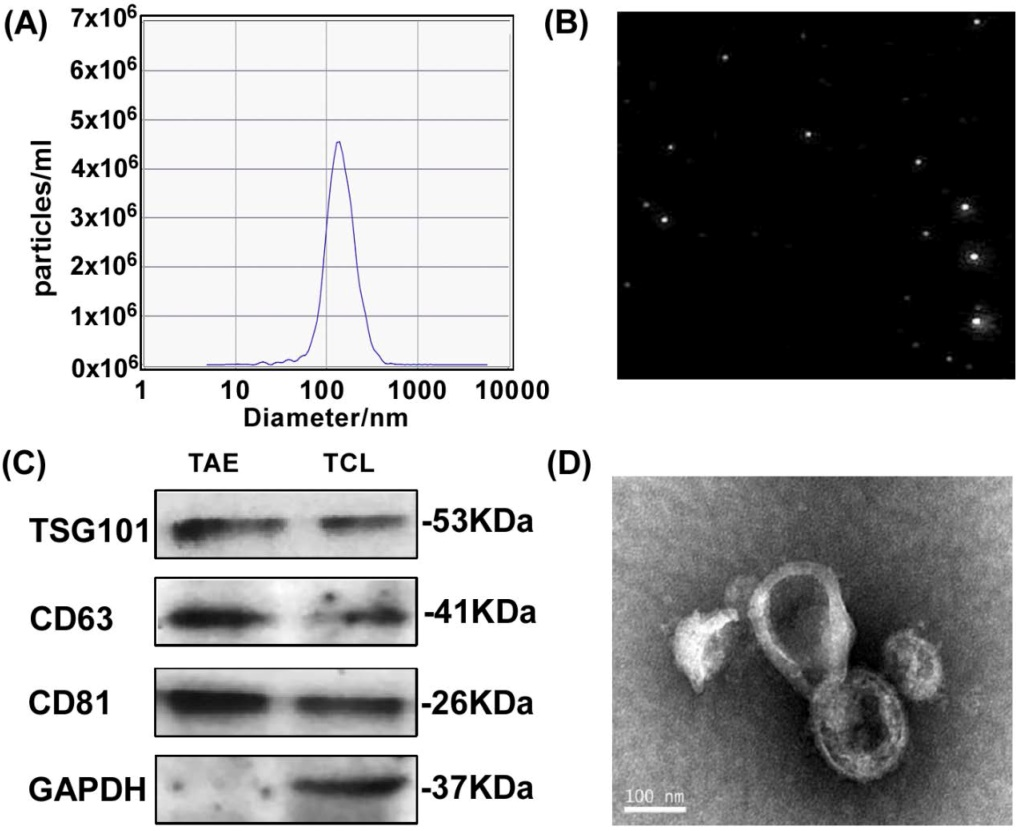
** Characterization and surface protein of A549 TAEs.** (**A-B**) The particle size measurement of exosomes. (**C**) Western blot analysis for detecting the expression of exosomal biomarkers and cellular protein in A549 TAEs. Total protein (20 μg) was loaded for A549 cell lysates and TAEs. (**D**) Transmission electron microscopic image of A549 TAEs (arrowheads, scale bar = 100 nm). Experiments were repeated three times in triplicate each time (n = 3).

To test the feasibility of A549 tumor-associated exosomes (A549-TAEs) as a source of antigens for DC-mediated antitumor immunity in lung tumors, we first measured the size of TAEs using nanoparticle tracking analysis. The results showed that the size of exosomes of A549 cells was 135.5 nm and the concentration of exosome was 5.6 × 10^7^/mL at 12000 times dilution (Fig. 1A-B), which was quite stable without size change for 7 days. We next characterized TAEs with exosomal biomarker proteins including transmembrane protein CD63, TSG101 and CD81 (Fig. 1C). The TEM image (Fig. 1D) revealed that the TAEs were dispersed as individual particles with a well-defined spherical shape and homogeneously distributed. The yield of TAEs was about 8-10 µg of protein per million A549 cells in 24 hours, which is similar to other tumor cells.

### TAEs promoted the maturation of DCs and antigen-presenting capability

It was reported that exosomes derived from tumor cells were highly enriched with tumor-associated antigens and a variety of immune-related proteins such as major histocompatibility complex (MHC) molecules 20 and heat shock proteins (HSPs) 21. The heat shock protein 70 (HSP70) was highly expressed in lung cancer cells and tissues 22, and was closely related to the growth and metastasis of lung cancer. Carbohydrate antigen 125 (CA125) was also a specific protein for clinical detection of lung cancer, so we further detected the expression of HSP70 and CA125 in TAEs. Strikingly, similar levels of HSP70 and CA125 expression were detected in TAEs and TCLs (supplementary data Fig. S1). Therefore, TAEs could act as the antigen of DC vaccines against lung cancer, and then trigger specific T-cell immune response to play an anti-tumor effect. Studies have shown that lung cancer cell-associated antigens stimulate dendritic cells to produce exosomes carrying lung cancer antigens 13, which can effectively activate CD4^+^ T and CD8^+^ T lymphocytes to produce strong anti-tumor immune response.

**Figure 2:**
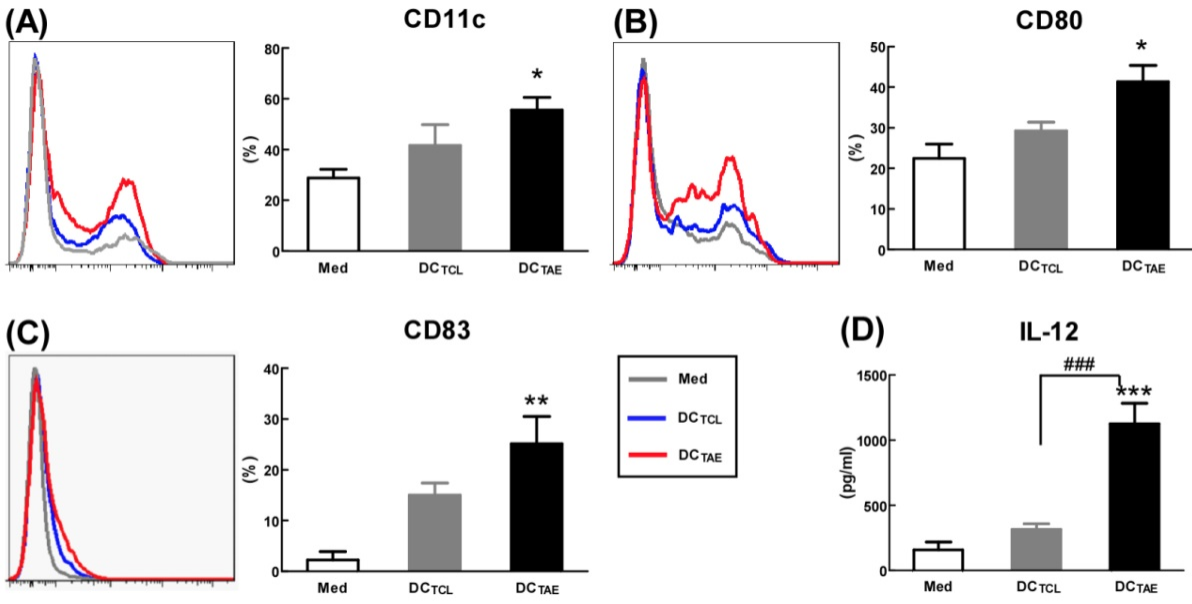
** The effect of TAEs on human monocyte-derived dendritic cells (DCs) maturation.** Monocyte-derived DCs were generated, as described in the methods section and were cultured with TAEs or TCLs (20 μg/ml) for 24 h. The expressions of CD11c (**A**), CD80 (**B**) and CD83 (**C**) on DCs were measured using flow cytometry. The productions of IL-12 (**D**) in culture supernatants were measured using ELISA. Bars shown are mean ± SE (n = 3-4), and differences between medium and other groups are determined using one-way ANOVA analysis. *: p < 0.05; **: p < 0.01; ***: p < 0.001. Differences between two different groups are statistically different, ###: p < 0.001.

Mature DCs express abundant co-stimulatory molecules, such as CD80 and CD83, which provide important signals for triggering downstream T lymphocyte activation 23. In the present study, TCL slightly induced CD83 and CD80, consistent with our previous observation 19. Notably, TAEs augmented CD80 by about 2 folds, CD83 by about 5 folds, suggesting that TAEs were more potent than TCLs alone to promote DC maturation (Fig. 2A-C). In addition to up-regulating DC maturation markers, TAEs also induced cytokine production. Our study showed that TCL doubled the production of IL-12, while TAEs dramatically increased IL-12 by over 5 folds (Fig. 2D). The superiority of maturation of DCs was probably because TAEs were easily taken up by DC 24. Previous study showed that the cellular uptake of soluble antigen was not sufficient and failed to induce full maturation of DCs 25. However, effective antigen uptake significantly elevated the maturation of DCs 26, which should be a key mechanism contributing to the superiority of TAEs. Overall, TAEs robustly induced the maturation of DCs and the production of type I IFNs.

**Figure 3:**
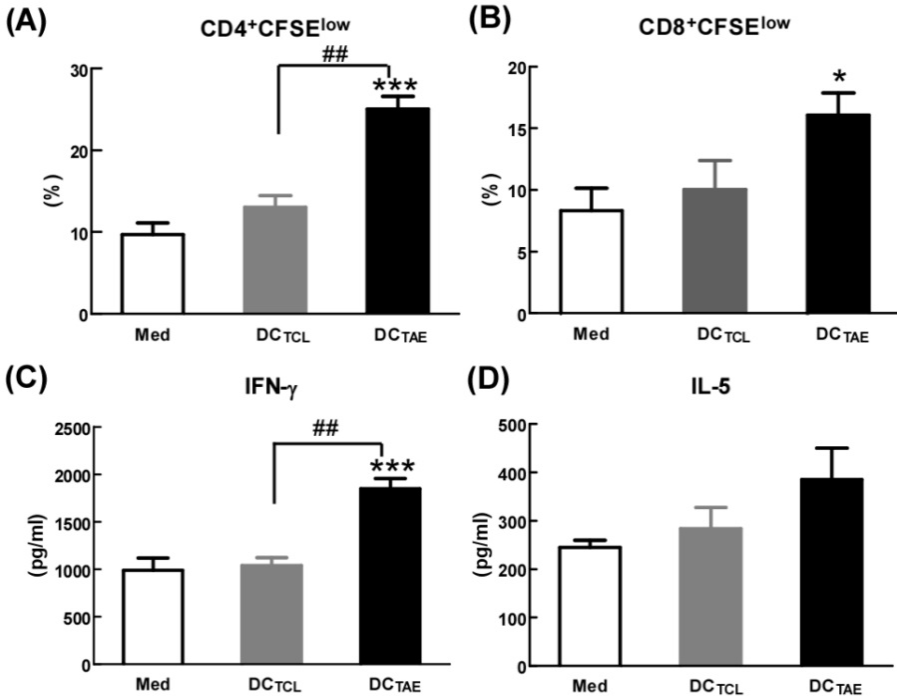
** The effect of TAEs on human monocyte-derived DC antigen presentation.** (**A-B**) DCs were treated with TAEs or TCLs (20 μg/ml) for 24 h, followed by co-culturing with CFSE-labeled CD8^+^ or CD4^+^ T cells for 48 h as described in Methods. MHC I and II antigen presentation were determined by measuring DC-primed CD8^+^ and CD4^+^ T cell proliferation (defined as CFSE^low^), respectively. The productions of IFN-γ (**C**) and IL-5 (**D**) in culture supernatants were measured using ELISA. Bars shown are mean ± SE (n = 3-4), and differences between medium and other groups are analyzed using one-way ANOVA analysis. *: p < 0.05; ***: p < 0.001. Differences between two different groups are statistically different, ##: p < 0.01.

By seeing DC maturation, we further evaluated their effect on DC-mediated MHC class I and II antigen presentation. PBMCs were treated with TAEs or TCLs for 24 h, followed by co-culture with CD4^+^ or CD8^+^ T lymphocytes for 48 h. The results showed that DCs treated by TAEs not only effectively elicited specific CD4^+^ T cell and CD8^+^ T cell proliferation (Fig. 3A-B), but also increased the productions of IL-5 and IFN-γ (Fig. 3C-D). Therefore, TAEs significantly enhanced DC-mediated MHC class II and MHC class I cross presentation. It was noteworthy that TCL-treated DC did not elicit specific CD8^+^ T cell proliferation (Fig. 3B), nor did they induce IFN-γ production by CD8^+^ T cells. Lack of IFN-γ production by CD8^+^ T cells suggested that CD8^+^ T cell-mediated CTL response could be limited 27. As is known, antigen-specific CD8^+^ T cell activation strongly depends on IL-12, a pre-inflammatory cytokine derived from DCs and macrophages 28. In the present study, the TCL did not elicit IL-12 production in DCs (Fig. 2D), which should be the mechanism causing limited CD8^+^ T cell activation. Furthermore, TCL-treated DC failed to elicit specific CD4^+^ T cell proliferation either (Fig. 3A). Nevertheless, TAEs significantly induced DC maturation and enhanced DC-mediated MHC class antigen presentation.

### DC_TAE_ vaccines potently induced anti-cancer immunity and suppressed cancer growth in mice

**Figure 4:**
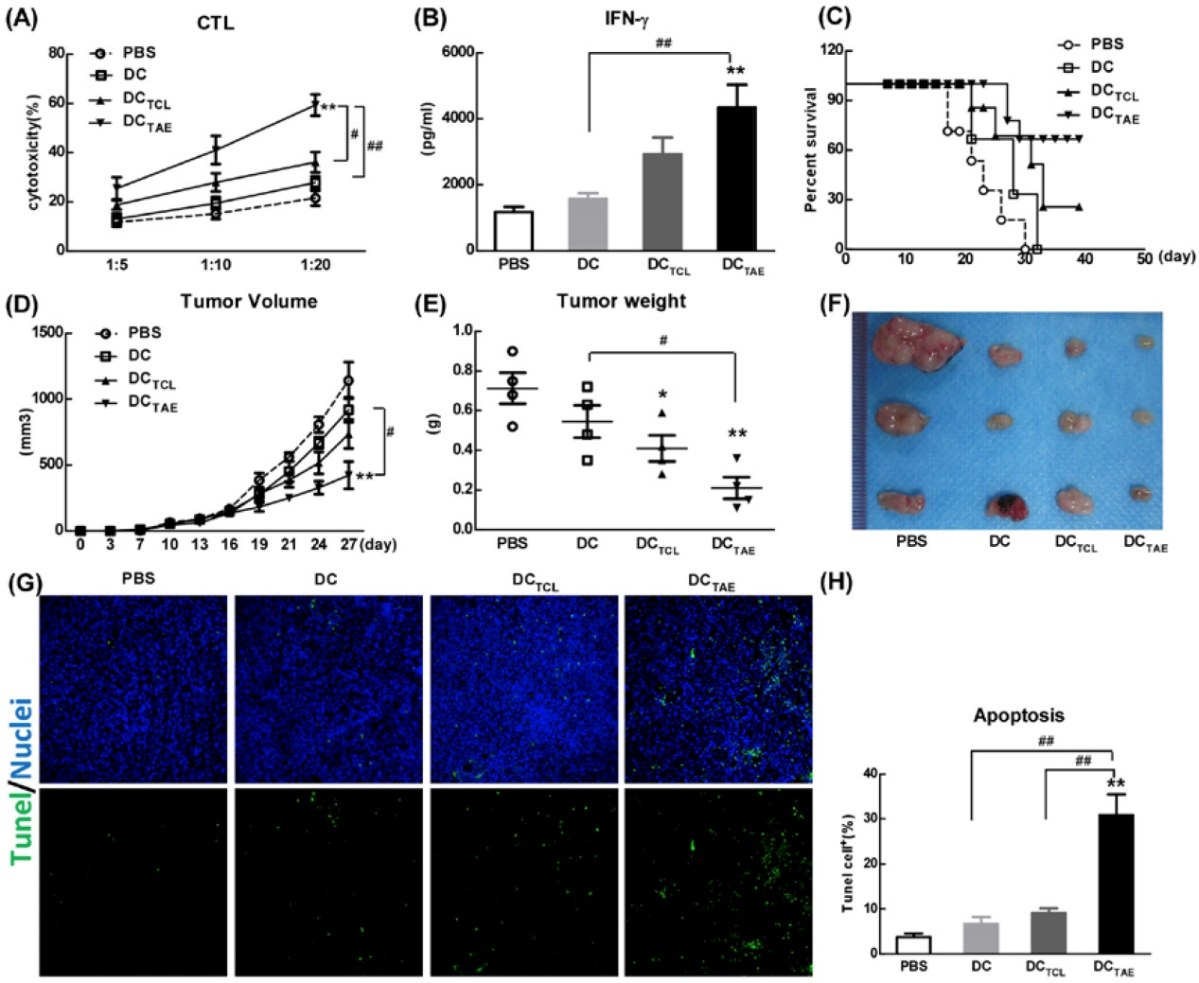
** DC_TAE_ vaccines induce tumor-specific immune responses in mice.** (**A** and **B**) Six-week C57BL/6 mice were i.v. immunized with different vaccines at day 0 and 7 as previously described. (A) Seven days after last immunization, total splenocytes were re-stimulated with LLC tumor cell lysates as described in Methods in the presence of IL-2 for 72 h, and then co-cultured with target cells (LLC cells) at different ratios of effector cells to target cells (E:T ratio) for another 4 h. Tumor-specific *in vitro* CTL response was analyzed using nonradioactive cytotoxicity assay, and the production of IFN-γ (B) in culture supernatants was measured using ELISA. Tumor-bearing mice were immunized with different vaccines once a week for 3 weeks from day 7 after tumor implantation. The survival rate (**C**) and tumor volume (**D**) were monitored every 2-3 days. (**E-F**) Measurement of subcutaneous tumor weight at 35 days after inoculation. Cell apoptosis in tumor tissue cryostat sections was detected using TUNEL assay (**G**), and the percentage of apoptotic cells (TUNEL^+^) was quantified using image J software (**H**). Bars shown are mean ± SE (n = 5-6), and differences between PBS and other groups are determined using one-way ANOVA analysis. **: p < 0.01. # Differences between two different groups are statistically different, #: p < 0.05; ##: p < 0.01.

The capability to elicit CTL response has long been considered as a major capability of therapeutic cancer vaccines 29. However, A549 was a human lung adenocarcinoma cell line, which could not be applied to construct tumor models in mice, so we used a mouse lung adenocarcinoma cell (Lewis lung carcinoma cells, LLC) *in vivo* research. In this study, we extracted LLC tumor-associated exosomes (LLC-TAEs) from the supernatant of LLC cells culture medium by ultracentrifugation. LLC-TAEs showed similar results in bone marrow-derived DC (BMDC) of mice (data not shown). Therefore, we first evaluated tumor-specific CTL responses induced by different cancer vaccines in healthy mice. The results showed that DC alone failed to induce anti-cancer CTL responses. However, DC_TAE_ vaccines effectively elicited tumor-specific CTL responses (Fig. 4A). In addition, TAEs robustly increased tumor-specific IFN-γ over 3 folds (Fig. 4B), indicating an enhanced Th1 response contributing to the augmented CTL responses. IFN-γ, as a Th1 signature cytokine, not only is essential for developing anti-cancer CTL responses, but also participates in tumor immunologic surveillance 30. Hence, DC_TAE_ vaccines induced robust antitumor immune responses, which could be attributable to enhanced DC maturation and MHC I antigen presentation by TAEs.

The anti-tumor effect of different vaccines was further investigated in tumor-bearing mice after immunization with 3 dosages of different vaccines. The results showed that DC alone did not suppress the tumor growth *in vivo*, while either DC_TAE_ or DC_TCL_ led to partial tumor regression. Notably, DC_TAE_ vaccines significantly prolonged survival time and increased survival rate in mice compared with the DC and PBS treatment groups (Fig. 4C), and DC_TAE_ vaccines were more potent than DC_TCL_ vaccines to suppress the tumor growth in mice (Fig. 4D). The results of TUNEL assay demonstrated that DC_TCL_ vaccines slightly induced tumor apoptosis, while DC_TAE_ vaccines dramatically increased cell apoptosis in tumor tissues as compared with other treatments (Fig. 4G). These data demonstrated that DC_TAE_ vaccines robustly induced tumor-specific CTL response and suppressed tumor growth *in vivo*. These data suggested that CD8**^+^** T cells act as principal effectors in local anti-tumor immune responses. Actually, the superior antitumor activity of DC_TAE_ vaccines was predominantly mediated by tumor-specific CD8^+^ CTLs. Previous studies showed that TAEs were more potent than TCLs to induce tumor-specific CTL responses in mice 16. Although DC_TAE_ vaccines significantly inhibited the growth of tumors, tumors were not completely eradicated, whose cause might be the existence of cancer stem cells 31, thus further confirmed the difficulty of treating lung tumors at present. In addition, delivery strategies and pathways may also affect the immunotherapeutic effect of DC_TAE_ vaccines 32, so there is much room for optimization.

### DC_TAE_ vaccines increased tumor-infiltrating CD8^+^ T cells in tumor microenvironment

**Figure 5:**
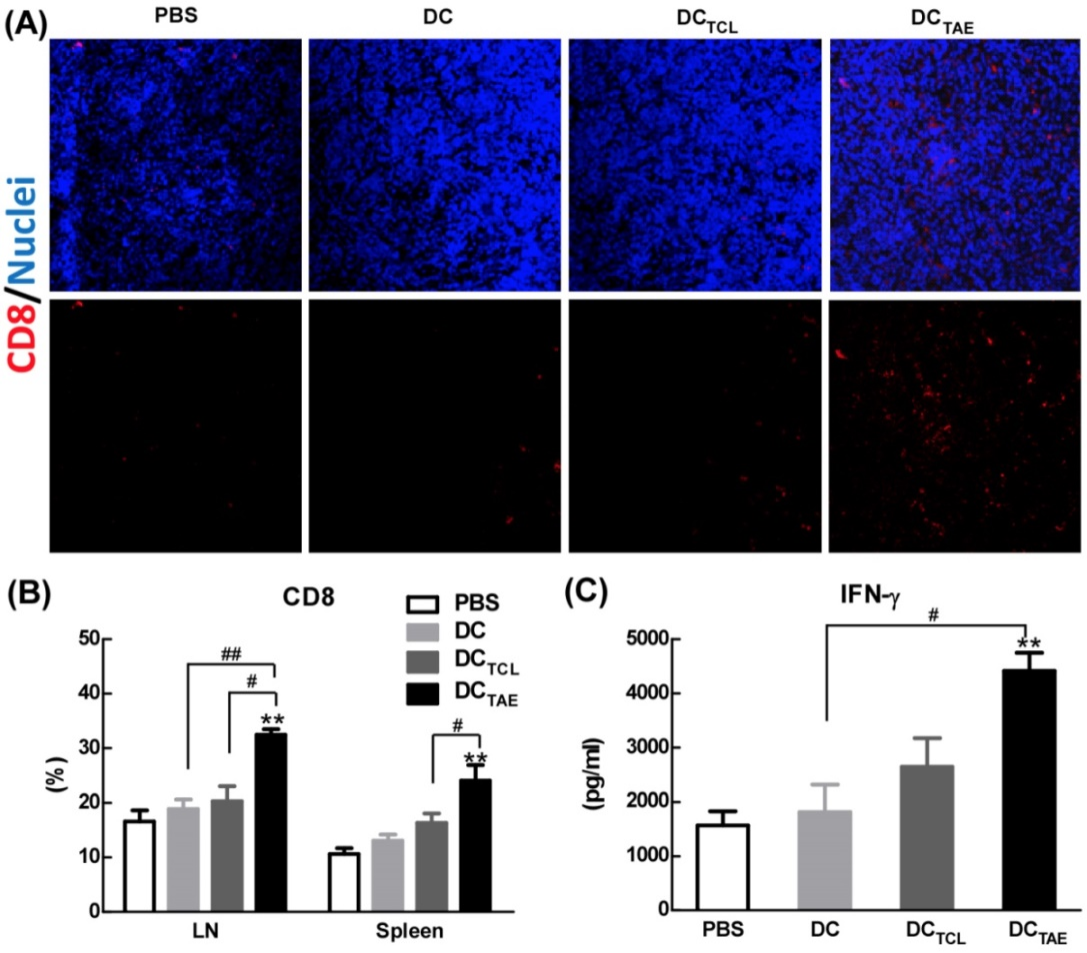
** The effect of cancer vaccines on CD8^+^ T cells in tumor bearing mice.** C57BL/6 tumor bearing mice were established as described in Fig. 4, and i.v. injected with different vaccines on day 7, 14, and 21 post tumor inoculation. One day after 3rd injection, tumors were dissected and then snap frozen. Frozen section of tumor tissues were labeled with PE-anti-mouse CD8 (**A**) as described in Section 2, and immunofluorescence images were recorded using a confocal microscopy. Splenocytes and TDLNs were isolated and labeled with PE-anti-mouse CD8, and the percentages of CD8^+^ T cells (**B**) in total splenocytes were measured using flow cytometry. Some splenocytes were re-stimulated with LLC TCLs *in vitro*, and the productions of IFN-γ were measured using ELISA (**C**). Images shown represent the data from 5 mice/group. Bars shown are mean ± SE (n = 4-5), and the differences among groups were analyzed using one-way ANOVA analysis followed by Tukey's post test. **: p < 0.01. # Differences between two different groups are statistically different, #: p < 0.05; ##: p < 0.01.

Vaccine-induced tumor regression and tumor cell apoptosis are generally mediated by CD8**^+^**T cell-mediated CTL responses, therefore we next investigated the presence of CD8**^+^**T cells in the tumor microenvironment. In addition, the recruitment of CD8^+^ T cells into the tumor microenvironment has been reported as a key parameter directly correlated with cancer prognosis. In the present study, immunization with DC without Ag barely recruited CD8**^+^** T cells into tumor tissues. However, DC_TCL_ vaccines modestly increased tumor-infiltrating CD8^+^ T cells (Fig. 5A), consistent with our previous observation 19. More importantly, DC_TAE_ vaccines robustly increased amount of CD8**^+^**T cells in tumor tissues (Fig. 5A), consistent with the results of TUNEL assay described previously. The strong Th1 immunity and CD8^+^ T cell activation induced by TAEs could be attributable to enhanced DC maturation (Fig. 2) and reduced immunosuppressive cells, such as Tregs. These data demonstrated that DC_TAE_ vaccine evoked anti-tumor immune responses *in vivo*.

In addition to recruiting CD8**^+^** T cells into tumor microenvironment, DC_TAE_ vaccines significantly increased the percentage of CD8**^+^** T cells compared with the DC and PBS treatment groups in spleen and TDLNs (Fig. 5B). The production of tumor-specific IFN-γ by splenocytes in tumor-bearing mice was also significantly enhanced by DC_TAE_ vaccines (Fig. 5C), a functional parameter of the T cell immune response 33, which could further promote the anti-tumor cytotoxicity of CD8^+^ T cells. Overall, DC_TAE_ vaccines significantly increased CD8^+^ T in tumor tissues and in peripheral lymphoid organs, thereby contributing to enhanced anti-tumor immune responses. These findings revealed that DC_TAE_ vaccines could trigger a strong antitumor immune response and reshape the tumor microenvironment in tumor-bearing mice. Overall, DC_TAE_ not only robustly induced tumor-specific CD8^+^ T cell activation, but also promoted their migration into the tumor environment, which consequently lead to effective tumor regression.

### DC_TAE_ vaccines improved tumor immune microenvironment in tumor-bearing mice

**Figure 6:**
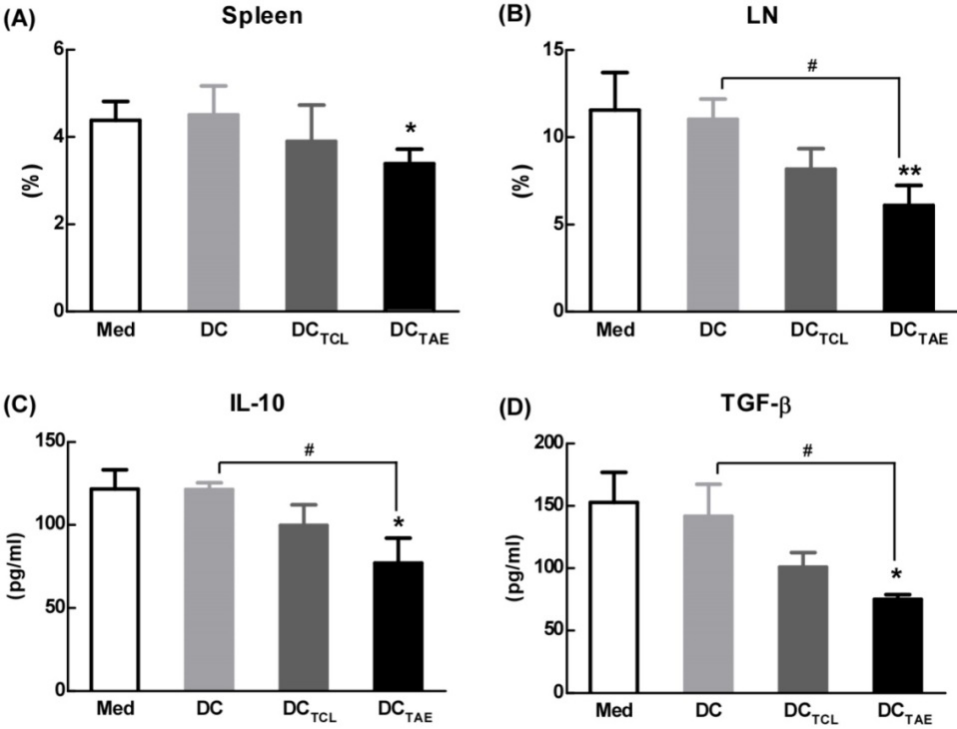
** The effect of different vaccines on Treg in tumor-draining lymph nodes (TDLNs).** C57BL/6 tumor bearing mice were established as described in Fig. 4, and i.v. injected with different vaccines on day 7, 14, and 21 post tumor inoculation. Analysis of CD4^+^FoxP3^+^CD25^+^ Treg cells in tumor tissues from tumor-bearing mice treated with DC_TAE_, DC_TCL_, DCs, or PBS in spleen (**A**) and TALN (**B**). The productions of TGF-β (**C**) and IL-10 (**D**) in serums from treated mice with measured using ELISA. Bars shown are mean ± SE (n = 4-5), and the differences among groups were analyzed using one-way ANOVA analysis followed by Tukey's post test. *: p < 0.05; **: p < 0.01. # Differences between two different groups are statistically different, #: p < 0.05.

Tumor-draining lymph nodes (TDLNs) are critical site for generating tumor-specific immune responses 34. Unfortunately, TDLNs are directly influenced by tumor-derived immunosuppressive factors, which lead to impaired DC maturation. Previous studies have shown that DC dysfunction contributed to the differentiation of regulatory T cells (Tregs) and myeloid-derived suppressor cells (MDSCs), which further exacerbated immunosuppression in tumor microenvironment and facilitated tumor progression 35-36. Tregs in the tumor microenvironment, especially in TDLNs, play a key role in tumor progression and tumor immune escape 18, 37. Although the study of subcutaneous transplantation of lung cancer model used in this study might not exactly reproduce the true immunosuppressive effect of tumor immune microenvironment in lung cancer patients, the immunization of DC_TAE_ vaccines not only significantly decreased the percentage of Tregs in the spleen, but also lowered the percentage of Tregs in TDLNs by 30-40% as compared with the other groups (Fig. 6A-B), suggesting that DC_TAE_ vaccines ameliorated immunosuppression in tumor-bearing mice. Notably, although DC_TCL_ vaccines slightly decreased the percentage of Tregs in TDLNs, they did not affect Tregs in spleen (Fig. 6A-B). These results suggested that the TCL-induced DCs might not be fully mature, which therefore failed to modulate Tregs in spleen. Taken together, immunization of DC_TAE_ vaccines effectively decreased Tregs in the spleen and TDLNs, which would consequently favor the establishment of antitumor immune responses. Consistently, analysis of serum cytokines from DC_TAE_ vaccines treated mice indicated a dramatic reduction in immunoinhibitory IL-10 and transforming growth factor-β (TGF-β) (Fig. 6C-D). These results demonstrated that DC_TAE_ vaccines altered the immune milieu from immunoinhibitory to immunostimulatory and is critical for the prognosis of lung tumor 38. These findings suggested that DC_TAE_ vaccines could trigger a strong antitumor immune response and improve the tumor microenvironment in tumor-bearing mice.

### TAEs down-regulated PD-L1 expression on DCs and TAE-pulsed DCs reduced Tregs population *in vitro*

**Figure 7:**
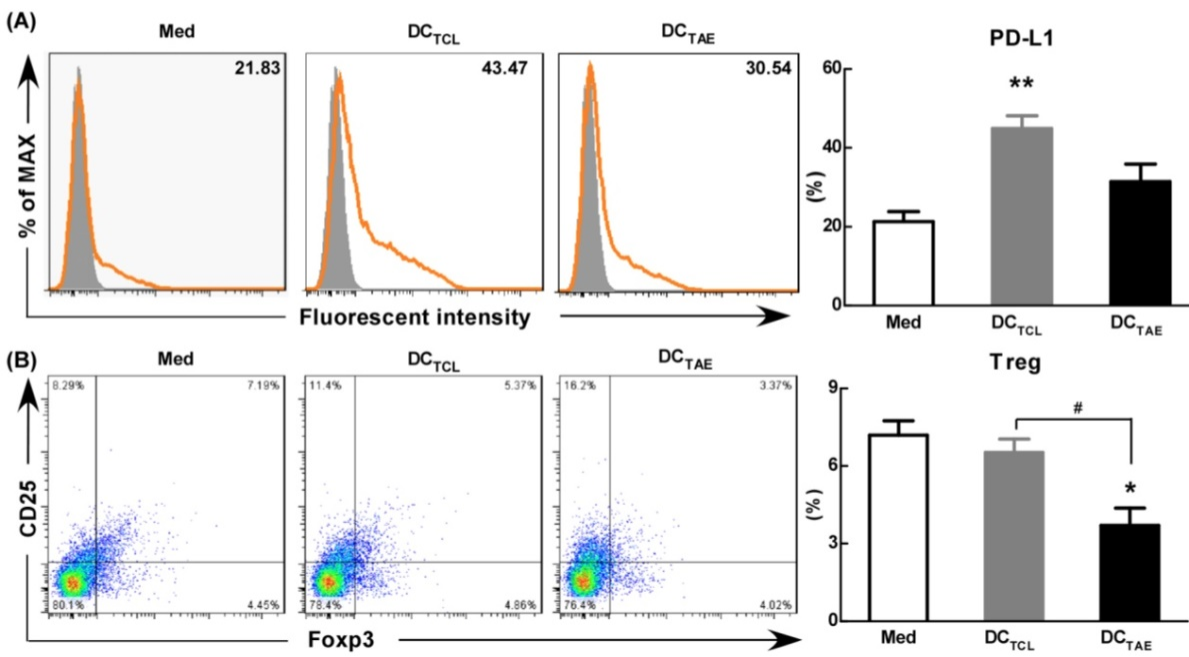
** The effect of TAEs on the expression of PD-L1 and Tregs *in vitro*. Monocyte-derived DCs were generated, as described in the methods section and were cultured with TAEs or TCLs (20 μg/ml) for 24 h.** The expression of PD-L1 on DCs was measured using flow cytometry (**A**). Some DCs were co-cultured with T cells at rate of 1:10. The expression of CD4^+^FoxP3^+^CD25^+^ on T cells was measured using flow cytometry (**B**). Bars shown are mean ± SE (n = 3), and the differences among groups were analyzed using one-way ANOVA analysis followed by Tukey's post test. *: p < 0.05; **: p < 0.01. # Differences between two different groups are statistically different, #: p < 0.05.

As is known, the maturation status of DCs is crucial for triggering downstream T lymphocyte activation and anti-tumor immune responses. During activation, DCs up-regulate inhibitory molecules PD-L1 that can bind PD-1 on activated T cells and inhibit T cell activation 39. Curiel et al. found that inhibitory cytokines such as IL-10 and VEGF in tumor microenvironment increased the expression of PD-L1 in DCs 40, and the DCs with high expression of PD-L1 were dysfunctional 41. In addition, our previous study confirmed that tumor supernatants caused dysfunction of DCs 19 and increased the expression of PD-L1. In the present study, we observed the expression of PD-L1 was 20-25% in immature DCs (Fig. 7A). However, DC_TCL_ have increased the expression of PD-L1 over 2 folds (Fig. 7A). It was worth noting that DC_TAE_ reduced the expression of PD-L1 compared with DC_TCL_, which might explain why Tregs down-regulated after DC_TAE_ treatment *in vivo* (Fig. 6A-B).

Moreover, DC_TCL_ barely decreased the percentage of Tregs *in vitro*, however, DC_TAE_ markedly reduced the population of Tregs (Fig. 7B). These results suggested that DCs induced by TAEs might be fully mature (Fig. 2), therefore affected to modulate downstream Tregs (Fig. 7B). As is known, PD-1/PD-L1 pathway interactions inhibit the functions and proliferation of activated T lymphocytes by direct contact or by changing the immune microenvironment 42-43. When blocking the PD-L1 signal of DCs in tumor microenvironment, a more effective T cell immune response was induced 44. Some studies have investigated the interruption of PD-1/PD-L1 pathway in non-small cell lung cancer and has shown promising results 45. Hence, TAEs effectively reduced PD-L1 expression of DCs, which should be a key mechanism contributing to superior anti-tumor immune responses of DC_TAE_. However, the difference of PD-L1 regulation between TAEs and TCLs was not clear, which might be related to the functional protein in TAEs and TCLs. At present, proteomics has been studied in many diseases, including many malignant tumor diseases 46. Therefore, we analyzed the differential proteomics of TAEs and TCLs of non-small cell lung cancer cell line by protein mass spectrometry. Although TAEs and TCLs had many common proteins, they also had some specific differential proteins respectively (data not shown). In addition, these differential proteins were rich in multiple functional pathways by Gene Ontology, such as cell adhesion molecule, extracellular matrix, signal receptor, where their specific regulatory functions need to be further verified. Overall, TAEs effectively down-regulated PD-L1, promoted DC maturation and reduced Tregs.

## Conclusion

Currently, antitumor effect of DC vaccine-based immunotherapy remains unsatisfactory due to poor immunogenicity of tumor antigens. In the present study, we extracted TAEs from the supernatant of tumor cell culture medium, and found TAEs superior over TCLs as antigens of DC vaccines. Our data showed that TAEs were more potent than TCLs to promote DC maturation and enhance MHC cross presentation, which directly contributed to more robust CTL response. More importantly, TAEs reduced the expression of PD-L1 of DCs, contributing to down-regulated population of Tregs *in vitro*. Moreover, DC_TAE_ remarkably suppressed the tumor growth and prolonged survival time *in vivo*, due to participance of CD8^+^ T cells and decreased Tregs in TDLNs and spleen. These data revealed that TAEs could serve to improve vaccine-elicited immunotherapy by triggering stronger DC-mediated immune responses and effectively abrogating immunosuppression in the tumor immune microenvironment.

## Supplementary Material

Supplementary figure.
