# Supplementary material for: Tumor Cell-associated Exosomes Robustly Elicit Anti-tumor Immune Responses through Modulating Dendritic Cell Vaccines in Lung Tumor
Source: Int J Biol Sci. 2020 Jan 14;16(4):633–43. doi: 10.7150/ijbs.38414 (PMC6990923; doi:10.7150/ijbs.38414)
Supplement: Supplementary file 1 — Supplementary figure. [file ijbsv16p0633s1.pdf]

Supplementary data

Fig.S1

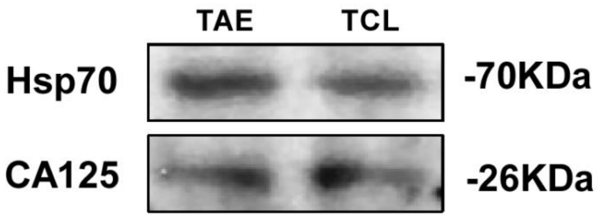

Fig. S1. The enrichment of tumor-associated antigens of tumor-associated exosomes. Western blots for examining levels of lung-specific antigens in A549 TAEs. Total protein (20μg) was loaded for A549 TCLs and TAEs to measure the expression of heat shock protein 70 (Hsp70) and CA125. A549 TCLs and TAEs loading used are noted in the image.
